# An Evaluation of the Cytocompatibility of Endodontic Bioceramics in Human Periodontal-Ligament-Derived Cells

**DOI:** 10.3390/jfb15080231

**Published:** 2024-08-19

**Authors:** Asuka Aka, Takashi Matsuura, Atsutoshi Yoshimura

**Affiliations:** Department of Periodontology and Endodontology, Nagasaki University Graduate School of Biomedical Sciences, Nagasaki City 852-8588, Nagasaki, Japan

**Keywords:** endodontic bioceramics, MTA Repair HP, MTA Flow White, Nishika Canal Sealer BG multi, cytocompatibility, human periodontal-ligament-derived cells

## Abstract

The present study evaluated the cytocompatibility of three endodontic bioceramics in human periodontal-ligament-derived cells (hPDLCs): MTA Repair HP (HP), MTA Flow White (F), and Nishika Canal Sealer BG multi (BG). In addition, we also evaluated the effect of the powder–liquid (paste) ratio of F and BG on cytocompatibility. Discs of endodontic bioceramics (diameter = 8 mm, thickness = 1 mm) were prepared with HP, F, and BG. hPDLCs obtained from extracted teeth and cultured for three to five passages were used in the experiment. The prepared discs were placed at the bottom of a 48-well plate, seeded with hPDLCs at 100,000 cells/well, cultured for 7 or 28 days, and subjected to a 3-[4,5-dimethylthiazol-2-yl]-2,5 diphenyl tetrazolium bromide assay. hPDLCs cultured without any discs were used as a negative control (NC) group. Discs made of F or BG mixed in three different consistencies were also used in this experiment. The absorbance values at days 7 and 28 were high in the order of HP > NC > BG > F. Furthermore, F or BG with higher consistency showed higher absorbance values. MTA Repair HP had the highest cytocompatibility among the three materials. Furthermore, it also showed that higher consistency improved cytocompatibility.

## 1. Introduction

One of the keys to successful root canal therapy is the proper closure of the prepared root canal cavity to prevent the entry of microorganisms and their irritants from the oral cavity into the root canal system and periapical tissue [1,2,3]. On the other hand, some endodontic materials, when in direct contact with periapical tissue, cause inflammation and the stimulation of sensory neurons that delay tissue healing and induce persistent postoperative pain [4,5]. Therefore, endodontic materials should have a good sealing ability and high biocompatibility.

Gutta-percha and conventional endodontic sealers such as epoxy-resin-based sealer have been used for root canal filling; however, they are difficult to use for root repair, such as in perforation repair, apical barrier, or root-end filling. Mineral trioxide aggregates (MTAs), such as ProRoot MTA, can be used for root repair, such as in perforation repair, apical barrier, or root-end filling, as an MTA induces hard tissue formation at the interface between the MTA and dentin and between the MTA and periapical tissue, resulting in high biocompatibility and sealing ability [6,7,8,9]. ProRoot MTA is thus the gold-standard root repair material; however, it has some disadvantages such as difficulty in handling, a long curing time, discoloration, and high solubility. Products that overcome these disadvantages are steadily being developed.

MTA Repair HP (HP; Angelus, Londrina, Brazil) is a calcium-silicate-based material that contains an organic plasticizer for easy handling [10]. It also contains calcium tungstate as a radiopacifier that does not cause tooth discoloration [10,11]. HP has higher push-out bond strength than conventional MTAs such as white MTA Angelus and there are no significant differences between them in curing time, radiopacity, solubility, or water absorption [11,12].

MTA Flow White (F; Ultradent Products Inc., South Jordan, UT, USA) is a Portland-cement-based material composed of a water-based gel and powders of tricalcium silicate and dicalcium silicate. The ratio of powder to gel is adaptable according to the required consistency, which ranges from thin to putty-like. MTA Flow with a thin consistency allows the easy insertion of the root canal using disposable syringes and 29-G NaviTip needles (Ultradent Products Inc., South Jordan UT, USA) [13]. It exhibits physicochemical, biological, and antimicrobial properties comparable to those of ProRoot MTA [13]. Furthermore, in studies, its discoloration was improved from that of its predecessor, MTA Flow, by changing the raw powder color from gray to white and the radiopacifier from bismuth oxide to tantalum oxide [13,14].

Nishka Canal Sealer BG multi (BG; Nippon Shika Yakuhin Co., Ltd., Yamaguchi, Japan) is a calcium silicate glass-based sealer. It is cured by mixing paste A, which contains fatty acids, with paste B, which contains magnesium oxide. The consistency can be adjusted, ranging from injectable paste to putty, by adding a powder containing calcium silicate glass and calcium hydroxide. BG contains bismuth hypocarbonate as a radiopacifier [15]. BG exhibits many desirable properties, including physicochemical stability, biocompatibility, sealing ability, and removability [15,16,17].

Many of these new products have been under development for less than a decade, so their characteristics have not been well studied. Thus, in the present study, we performed experiments to evaluate the cytocompatibility of the following three novel endodontic bioceramics for human periodontal-ligament-derived cells (hPDLCs): MTA Repair HP, MTA Flow White, and Nishika Canal Sealer BG multi (Table 1). The null hypothesis of this study was as follows:

**Null Hypothesis 1.**  *There is no significant difference in cytocompatibility between MTA Repair HP, MTA Flow White, Nishika Canal Sealer BG multi, and the negative control.*

In addition, the consistencies of F and BG can be adjusted by changing the powder–liquid (paste) ratio, so we also evaluated the effect of the powder–liquid (paste) ratio on cytocompatibility. This null hypothesis of this study is as follows:

**Null Hypothesis 2.**  *There is no significant effect of the powder–liquid (paste) ratio of F or BG on cytocompatibility.*

## 2. Materials and Methods

**Figure 1:**
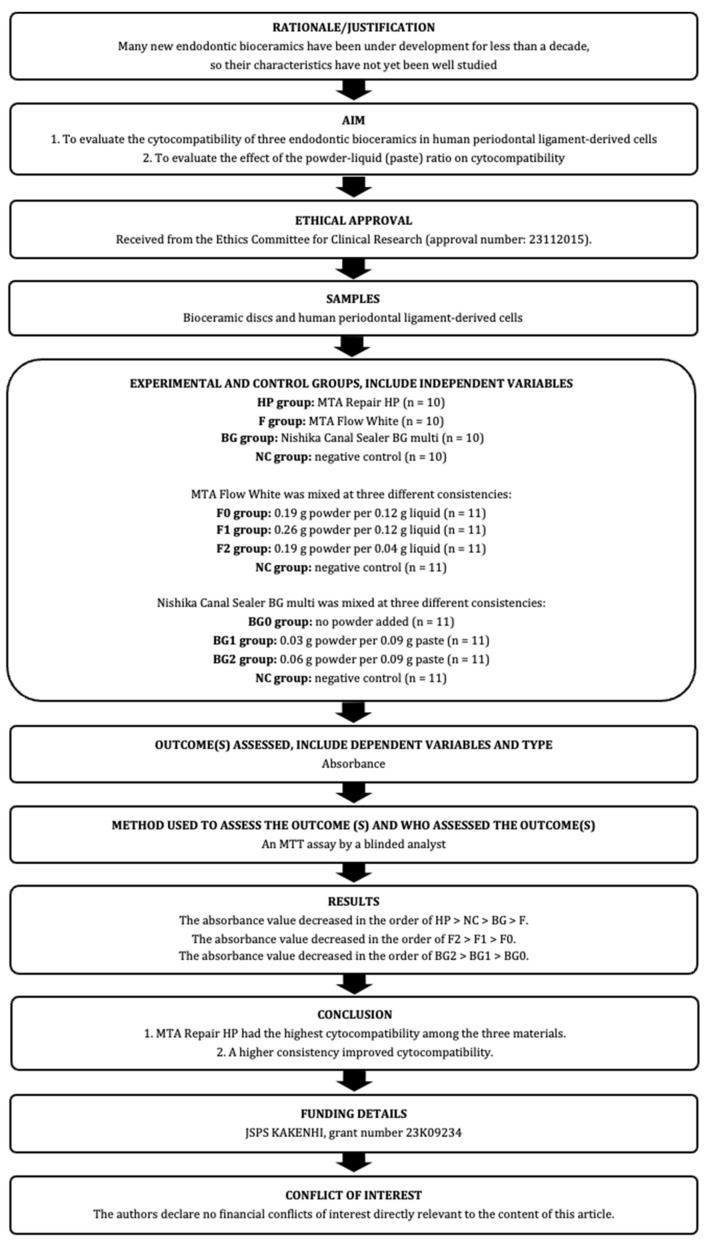
The PRILE 2021 flowchart of this study.

The manuscript of this laboratory study has been written according to Preferred Reporting Items for Laboratory studies in Endodontology (PRILE) 2021 guidelines [18,19]. The PRILE 2021 flowchart is shown in Figure 1 and the PRILE 2021 checklist is attached in Table S1. This study was approved by the Nagasaki University Hospital Clinical Research Ethics Committee on 22 November 2021 and 28 November 2023 (approval number: 21111512 and 23112015).

### 2.1. Human Periodontal-Ligament-Derived Cells (hPDLCs)

Teeth were obtained from patients who visited our hospital for tooth extraction and who provided verbal consent for this study. Healthy human premolars or third molars with no evidence of periodontitis, pericoronitis, or periapical pathology were included in this study. Periodontal ligament tissue was obtained using a scalpel (No. 11; Feather Safety Razor, Osaka, Japan) from the middle thirds of the roots of extracted teeth, seeded in 35 mm cell culture dishes, and cultured in Dulbecco’s Modified Eagle Medium (DMEM; FUJIFILM Wako Pure Chemical Corporation, Osaka, Japan) containing 10% fetal bovine serum (FBS; Life Technologies, Tokyo, Japan), 1% penicillin–streptomycin (PS; FUJIFILM Wako Pure Chemical Corporation), and 0.25 μg/mL amphotericin B (FUJIFILM Wako Pure Chemical Corporation) at 37 °C in a 5% CO_2_ incubator. After passaging, hPDLCs were cultured in DMEM containing 10% FBS and 1% PS. hPDLCs cultured for three to five passages were used in this study.

### 2.2. Preparation of Bioceramic Discs

HP, F, and BG were mixed according to the manufacturer’s instructions and loaded into a mold (inner diameter = 8 mm, thickness = 1 mm). F was prepared in soft consistency (powder–liquid ratio: 0.19 g powder per 0.12 g liquid). BG was mixed without the addition of powder. They were then placed in a 5% CO_2_ incubator at 37 °C and 100% humidity for 48 h (*n* = 10).

### 2.3. Cytocompatibility

**Figure 2:**
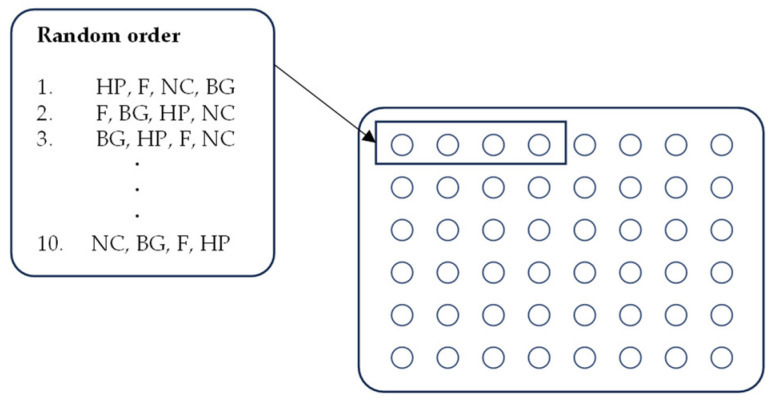
Random order was generated.

The prepared discs were placed at the bottom of 48-well plates. hPDLCs cultured without any discs were used as a negative control (NC) group. The random order was generated by AA with opaque sealed envelopes on which “HP”, “BG”, “F”, or “NC” was printed (Figure 2). Then, hPDLCs were seeded at a density of 100,000 cells/well and cultured in 500 μL DMEM containing 10% FBS and 1% PS. The 3-[4,5-dimethylthiazol-2-yl]-2,5 diphenyl tetrazolium bromide (MTT) assay was performed on days 7 and 28 of culture to measure cell metabolic activity using the MTT Cell Proliferation Assay Kit (Sigma-Aldrich, St. Louis, MO, USA). Then, plated discs were removed by AA and absorbance was measured by an analyst (T.M.) blinded to the order. Absorbance was measured using a microplate reader at a test wavelength of 570 nm and reference wavelength of 690 nm.

### 2.4. Effects of the Powder–Liquid (Paste) Ratio of F or BG on Cytocompatibility

F was mixed in three different consistencies: F0 (0.19 g powder per 0.12 g liquid), F1 (0.26 g powder per 0.12 g liquid), and F2 (0.19 g powder per 0.04 g liquid). BG was also mixed in three different consistencies: BG0 (no powder added), BG1 (0.03 g powder per 0.09 g paste), and BG2 (0.06 g powder per 0.09 g paste). Discs of F0, F1, F2, BG0, BG1, and BG2 were then prepared using the same procedure, hPDLCs were cultured on the prepared discs and the MTT assay was performed on days 7 and 28.

### 2.5. Statistical Analyses

Paired comparisons of the four groups (HP, F, BG, and NC) were performed using Welch’s *t*-test. The *p*-values were corrected with Bonferroni adjustment (*p* < 0.05) and the significance level was set at α = 0.05/6 = 0.008. Comparisons between the absorbance values on days 7 and 28 were performed using a paired *t*-test with *α* = 0.05.

Comparisons of groups F0 vs. F1, F2, or NC and comparisons of groups BG0 vs. BG1, BG2, or NC were performed using Welch’s *t*-test. The *p*-values were corrected with Bonferroni adjustment (*p* < 0.05) and the significance level was set at *α* = 0.05/3 = 0.016. Comparisons between the absorbance values on days 7 and 28 were performed using a paired *t*-test with *α* = 0.05. Data analyses were conducted with IBM SPSS statistics version 27.0 (IBM Inc., Chicago, IL, USA).

### 2.6. Sample Size

Pilot studies were conducted to calculate the sample size for each experiment. The data acquired in pilot studies are shown in Figures S1–S3. The sample sizes were calculated based on the results of the preliminary experiments. The sample size of the experiment to evaluate the cytocompatibility of HP, F, and BG was calculated to detect a difference between the F group (mean and standard deviation [SD]: 1.04 [0.25]) and the NC group (mean [SD]: 1.51 [0.06]) with a power of 90% at a 2-tailed significance level of 0.008. Ten teeth were used, with a 20% dropout rate. The sample sizes of the experiment to evaluate the effect of the powder–liquid (paste) ratio of F or BG on cytocompatibility were calculated to detect differences between the F0 group (mean [SD]: 0.01 [0.00]) and the NC group (mean [SD]: 1.26 [0.10]) and between the BG0 group (mean [SD]: 0.69 [0.43]) and the NC group (mean [SD]: 1.64 (0.12]) with a power of 90% at a 2-tailed significance level of 0.016. Eleven teeth were used, with a 20% dropout rate.

## 3. Results

**Figure 3:**
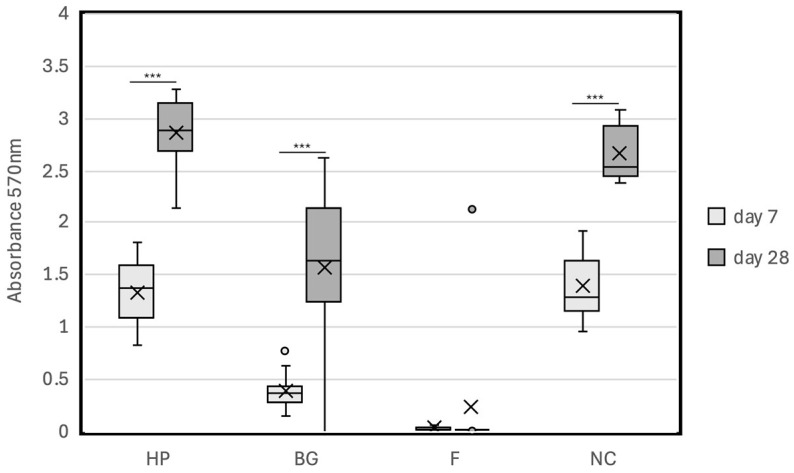
Cytocompatibility of endodontic bioceramics in hPDLCs after 7 days and 28 days of incubation with bioceramic materials. *** *p* < 0.001; 。: outlier; x: mean value; HP: MTA Repair HP; F: MTA Flow White; BG: Nishika Canal Sealer BG multi; NC: negative control.

We performed an MTT assay to evaluate the cytocompatibility of the novel endodontic bioceramics HP, F, and BG for hPDLCs. The results are shown in Figure 3 and Table 2 and Table 3. The absorbance (mean [SD]) values of the HP, BG, F, and NC groups on day 7 were 1.34 (0.33), 0.38 (0.20), 0.03 (0.02), and 1.39 (0.34), respectively, and those on day 28 were 2.87 (0.35), 1.58 (0.81), 0.23 (0.67), and 2.64 (0.32), respectively. There was no significant difference between the absorbance of the HP group and that of the NC group on days 7 and 28 (*p* = 0.712 and *p* = 0.159, respectively). However, the absorbance values of the BG and F groups were significantly lower than those of the NC and HP groups on days 7 and 28 (*p* < 0.008). There was no significant difference in absorbance between the BG and F groups on day 7, and on day 28, the absorbance of the BG group was significantly higher than that of the F group (*p* = 0.001). The absorbance value was high in the order of HP > NC > BG > F. The absorbance values of the HP, BG, and NC groups on day 28 were significantly higher than those on day 7 (*p* < 0.001). However, there was no significant difference in absorbance for the F group between days 7 and 28 (*p* = 0.401).

**Figure 4:**
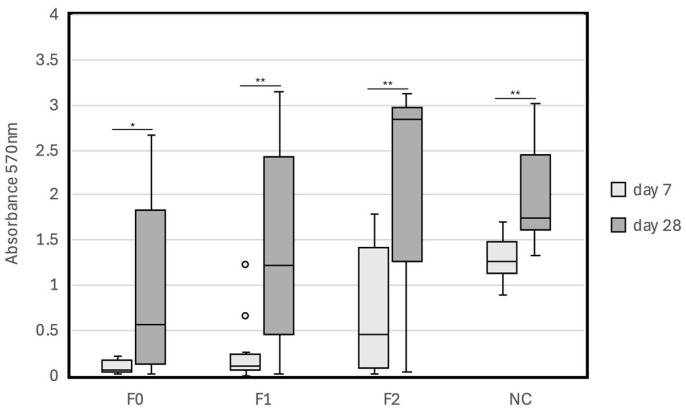
Cytocompatibility of MTA Flow White in different powder–liquid (paste) ratios with hPDLCs after 7 days and 28 days of incubation with MTA Flow White mixed in three different consistencies: F0 (0.19 g powder per 0.12 g liquid), F1 (0.26 g powder per 0.12 g liquid), and F2 (0.19 g powder per 0.04 g liquid). * *p* < 0.05; ** *p* < 0.01; 。: outlier; F: MTA Flow White; NC: negative control.

**Figure 5:**
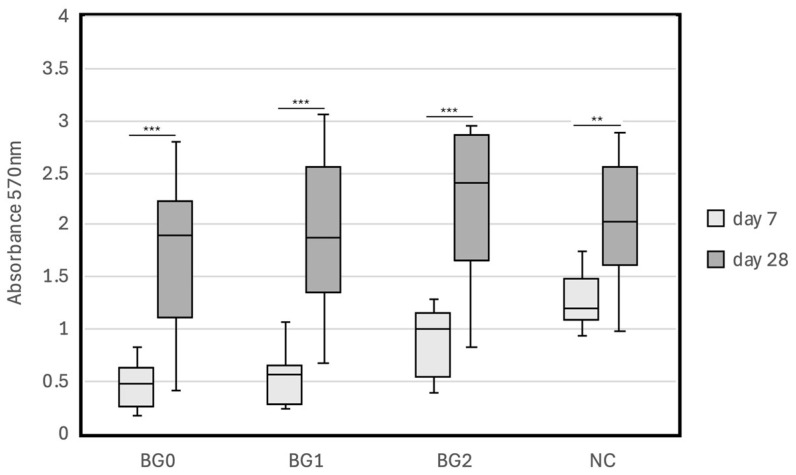
Cytocompatibility of Nishika Canal Sealer BG multi in different powder–liquid (paste) ratios with hPDLCs after 7 days and 28 days of incubation with Nishika Canal Sealer BG multi mixed in three different consistencies: BG0 (no powder added), BG1 (0.03 g powder per 0.09 g paste), and BG2 (0.06 g powder per 0.09 g paste). ** *p* < 0.01; *** *p* < 0.001; BG: Nishika Canal Sealer BG multi; NC: negative control.

Next, we evaluated the effect of the powder–liquid (paste) ratio on cytocompatibility. The results are shown in Table 4 and Table 5 and Figure 4 and Figure 5. The absorbance values of the F0, F1, F2, and NC groups on day 7 were 0.09 (0.08), 0.26 (0.37), 0.73 (0.72), and 1.28 (0.27), respectively, and those on day 28 were 1.02 (1.06), 1.44 (1.20), 2.18 (1.09), and 2.02 (0.61), respectively. The absorbance values of the BG0, BG1, BG2, and NC groups on day 7 were 0.45 (0.22), 0.55 (0.28), 0.87 (0.34), and 1.28 (0.28), respectively, and those on day 28 were 1.69 (0.79), 1.89 (0.84), 2.20 (0.82), and 2.04 (0.63), respectively. There was no significant difference between the absorbance values of the F0 and F1 groups on day 7 (*p* = 0.165); however, the absorbance values of the F2 and NC groups were significantly higher than that of the F0 group on day 7 (*p* = 0.015 and *p* < 0.001). There was no significant difference between the absorbance values of the BG0 and BG1 groups on day 7 (*p* = 0.398), and the absorbance values of the BG2 and NC groups on day 7 were significantly higher than that of the BG0 group on day 7 (*p* = 0.003 and *p* < 0.001, respectively). On day 28, a significant difference was observed only between the absorbance values of the F0 and NC groups (*p* = 0.015). The absorbance value decreased in the orders of F2 > F1 > F0 and BG2 > BG1 > BG0. The absorbance values on day 28 were significantly higher than those on day 7 in all groups (*p* < 0.05).

## 4. Discussion

Before starting this study, we evaluated the hard tissue inductivity of HP, BG, and F in hPDLCs. To induce hard tissue formation, hPDLCs should be cultured on discs for more than 20 days [20,21,22,23,24]. We therefore cultured hPDLCs for 28 days; however, the numbers of hPDLCs after 28 days of culturing in each group were too different to compare hard-tissue inductivity between groups. Some in vitro cytocompatibility studies had been conducted on the materials used in this study [10,12,13,15,16]. However, these studies had been conducted for short incubation periods, and there have been few studies on long-term cytocompatibility. Therefore, we evaluated the cytocompatibility of endodontic bioceramics with hPDLCs after 28 days of culturing.

The data of this study support the rejection of null hypothesis 1. The present study showed that HP had the highest cytocompatibility with hPDLCs among the three materials, which was consistent with previous studies [25,26]. In contrast, the cell metabolism of the F group on days 7 and 28 was significantly lower than that of the HP and NC groups. Pelepenko et al. reported that F has cytocompatibility similar to that of ProRoot MTA [13]. However, their experiment to evaluate F’s cytocompatibility may have lacked sufficient power to detect any difference because of the small sample size (*n* = 3). Furthermore, the cell culture period was 24 h, which may have been too short to evaluate the effect of the MTA on cells.

The absorbance values of the BG group on days 7 and 28 were also significantly lower than those of the HP and NC groups. Jo et al. reported that BG has superior cytocompatibility [16] in their experiments using the extracts of the materials. Such extracts are typically used for in vitro cytocompatibility studies on root canal sealers [27,28,29,30,31,32]. In experiments using extracts, suitable conditions are made available by changing the extraction time and the dilution ratio. However, the concentration of the extraction in those authors’ experiment may not have been identical to the actual concentration in vivo, and the concentration in some studies may have been too low.

Normally, endodontic sealers are compared with other types of sealers, not with root canal repair materials, such as MTAs. However, recently, bioceramic sealers, such as BG, have been used for root canal repair, including for perforation repair and apical plug, as with conventional MTAs. Therefore, it is necessary to compare bioceramic sealers with other root canal repair materials. Thus, we compared BG with HP and F in the present experiment using discs commonly used for in vitro cytocompatibility studies on MTAs. The results of this study may contribute to the selection of materials for root canal repair.

The data of this study also support the rejection of null hypothesis 2. This study showed that higher consistency improved the cytocompatibility of F and BG. Ma et al. reported that a shorter setting time resulted in lower cytotoxicity in MTAs [33]. F and BG, with their higher consistency, showed shorter setting times. This may be one of the reasons why the absorbances of F and BG with high consistency are higher than those with low consistency. Ma et al. showed that fresh materials have lower cytocompatibility than set materials [33]. In this experiment, discs were set for 48 h in a 5% CO_2_ incubator before cell culturing; however, for root canal repair, materials needed to be applied before setting. Therefore, the cytocompatibilities observed in this study may have been higher than in vivo biocompatibility. In a future study, we will perform in vivo biocompatibility studies for a more accurate prediction of how the materials will behave in the human body.

## 5. Conclusions

This study evaluated the in vitro long-term cytocompatibility of MTA Repair HP, MTA Flow White, and Nishika Canal Sealer BG multi on hPDLCs and revealed that MTA Repair HP had the highest cytocompatibility among the three materials. Furthermore, it also showed that higher consistency improved cytocompatibility in MTA Flow White and Nishika Canal Sealer BG multi. However, in vivo biocompatibility remains unclear; thus, further studies are required.

## Figures and Tables

**Table 1:** Materials used in this experiment.

Materials	Manufactures	Composition
MTA Repair HP	Angelus (Brazil)	Powder: calcium silicate, calcium aluminate, calcium oxide, calcium tungstate Liquid: purified water, plasticizer
MTA Flow White	Ultradent Products (USA)	Powder: tricalcium silicate, dicalcium silicate, calcium sulfate, etc. Gel: purified water, thickening agent
Nishika Canal Sealer BG Multi	Nippon Shika Yakuhin (Japan)	Paste A: fatty acids, bismuth bicarbonate, silicon dioxide Paste B: magnesium oxide, purified water, calcium silicate glass, silicon dioxide, etc. Powder: calcium silicate glass, calcium hydroxide

**Table 2:** Absorbance determined by the MTT assay after 7 days of incubation with bioceramic materials.

	1	2	3	4	5	6	7	8	9	10	Mean (SD)
HP	0.91	1.06	1.57	1.81	1.67	1.59	1.17	0.83	1.32	1.44	1.34 (0.33) ^a^
BG	0.15	0.41	0.43	0.63	0.76	0.15	0.27	0.33	0.27	0.43	0.38 (0.20) ^b^
F	−0.01	0.01	0.06	0.02	0.01	0.03	0.02	0.01	0.03	0.06	0.03 (0.02) ^b^
NC	1.28	1.13	1.27	1.90	1.54	1.67	1.05	0.96	1.92	1.22	1.39 (0.34) ^a^

HP: MTA Repair HP; F: MTA Flow White; BG: Nishika Canal Sealer BG multi; NC: negative control. Different lowercase letters indicate significant differences (*p* < 0.008).

**Table 3:** Absorbance determined by the MTT assay after 28 days of incubation with bioceramic materials.

	1	2	3	4	5	6	7	8	9	10	Mean (SD)
HP	3.12	3.16	2.93	2.81	2.85	2.66	2.15	2.52	3.18	3.28	2.87 (0.35) ^a^
BG	−0.08	2.22	1.38	2.42	1.90	1.21	1.34	0.85	1.94	2.62	1.58 (0.81) ^b^
F	0.01	0.02	−0.01	0.01	0.02	0.01	0.01	0.01	0.04	2.13	0.23 (0.67) ^c^
NC	2.47	2.99	2.62	2.79	2.45	2.45	2.45	2.39	3.07	3.08	2.64 (0.32) ^a^

HP: MTA Repair HP; F: MTA Flow White; BG: Nishika Canal Sealer BG multi; NC: negative control. Different lowercase letters indicate significant differences (*p* < 0.008).

**Table 4:** Absorbance determined by the MTT assay after 7 and 28 days of incubation with MTA Flow White in different powder–liquid ratios.

7 Days													
	1	2	3	4	5	6	7	8	9	10	11	Mean (SD)	*p*-Value
F0	0.03	0.02	0.01	0.05	0.07	0.22	0.01	0.18	0.22	0.06	0.14	0.09 (0.08)	reference
F1	0.00	0.23	1.22	0.67	0.04	0.05	0.06	0.10	0.04	0.22	0.25	0.26 (0.37)	0.165
F2	0.01	1.49	1.72	0.45	1.35	0.08	0.75	0.26	0.07	1.80	0.06	0.73 (0.72)	0.015
NC	1.37	1.20	1.70	1.54	1.58	0.90	1.26	1.05	0.90	1.21	1.41	1.28 (0.27)	<0.001
**28 Days**													
	**1**	**2**	**3**	**4**	**5**	**6**	**7**	**8**	**9**	**10**	**11**	**Mean (SD)**	***p*** **-Value**
F0	2.34	0.03	0.16	1.32	2.67	0.55	0.39	0.01	2.61	0.07	1.03	1.02 (1.06)	reference
F1	0.02	1.21	2.71	1.01	3.15	2.13	3.11	0.01	0.75	1.64	0.13	1.44 (1.20)	0.388
F2	0.04	3.04	2.92	1.43	2.55	1.09	3.14	3.09	2.88	0.97	2.85	2.18 (1.09)	0.02
NC	1.75	1.60	2.08	1.32	1.69	2.08	1.41	2.86	3.02	1.64	2.80	2.02 (0.61)	0.015

F0, F1, and F2: MTA Flow White mixed in three different consistencies—F0 (0.19 g powder per 0.12 g liquid), F1 (0.26 g powder per 0.12 g liquid), and F2 (0.19 g powder per 0.04 g liquid); NC: negative control. Significance level: <0.016.

**Table 5:** Absorbance determined by the MTT assay after 7 and 28 days of incubation with Nishika Canal Sealer BG multi in different powder–paste ratios.

7 Days													
	1	2	3	4	5	6	7	8	9	10	11	Mean (SD)	*p*-Value
BG0	0.62	0.39	0.62	0.48	0.83	0.58	0.62	0.22	0.16	0.28	0.18	0.45 (0.22)	reference
BG1	0.69	0.57	0.56	1.06	0.94	0.62	0.52	0.28	0.28	0.23	0.25	0.55 (0.28)	0.398
BG2	1.01	1.07	1.17	1.15	1.14	0.96	1.29	0.38	0.49	0.39	0.57	0.87 (0.34)	<0.01
NC	1.24	1.14	1.74	1.65	1.58	0.94	1.20	0.97	1.04	1.20	1.37	1.28 (0.28)	<0.001
**28 Days**													
	**1**	**2**	**3**	**4**	**5**	**6**	**7**	**8**	**9**	**10**	**11**	**Mean (SD)**	***p*** **-Value**
BG0	2.62	2.09	1.89	0.78	2.31	1.35	2.80	1.11	2.17	0.40	1.10	1.69 (0.79)	reference
BG1	2.78	1.87	2.35	1.03	2.97	1.69	3.06	1.88	1.75	0.68	0.80	1.89 (0.84)	0.563
BG2	2.93	2.95	2.81	0.83	2.40	2.75	2.96	2.16	2.18	1.01	1.18	2.20 (0.82)	0.157
NC	2.70	1.36	1.76	0.97	2.85	1.78	2.04	2.89	2.41	1.49	2.21	2.04 (0.63)	0.267

BG0, BG1, and BG2: Nishika Canal Sealer BG multi mixed in three different consistencies—BG0 (no powder added), BG1 (0.03 g powder per 0.09 g paste), and BG2 (0.06 g powder per 0.09 g paste); NC: negative control. Significance level: <0.016.

## Data Availability

The data presented in this study are available on request from the corresponding author.

## Supplementary Materials

The following supporting information can be downloaded at: https://www.mdpi.com/article/10.3390/jfb15080231/s1, Figure S1: Cytocompatibility of endodontic bioceramics in hPDLCs; Figure S2: Cytocompatibility of MTA Flow White in different powder–liquid (paste) ratios with hPDLCs; Figure S3: Cytocompatibility of Nishika Canal Sealer BG multi in different powder–liquid (paste) ratios with hPDLCs; Table S1: PRILE 2021 checklist; Table S2: Absorbance determined by the MTT assay after 7 days of incubation with bioceramic materials; Table S3: Absorbance determined by the MTT assay after 28 days of incubation with MTA Flow White in different powder–liquid ratios; Table S4: Absorbance determined by the MTT assay after 28 days of incubation with Nishika Canal Sealer BG multi in different powder–paste ratios.
