# Supplementary material for: An Evaluation of the Cytocompatibility of Endodontic Bioceramics in Human Periodontal-Ligament-Derived Cells
Source: J Funct Biomater. 2024 Aug 19;15(8):231. doi: 10.3390/jfb15080231 (PMC11355906; doi:10.3390/jfb15080231)
Supplement: Supplementary file 1 [file jfb-15-00231-s001.zip › jfb-3128531-supplementary.pdf]

Table S1: PRILE 2021 checklist

Checklist of items to be included when reporting laboratory studies in Endodontology*

Section/ Topic	Item Number	Checklist Items	Reported on page number
**Title**	1a	The Title must identify the study as being laboratory-based, e.g. "laboratory investigation" or "*in vitro*," or "*ex vivo*" or another appropriate term	1
	1b	The area/field of interest must be provided (briefly) in the Title	1
**Keywords**	2a	At least two keywords related to the subject and content of the investigation must be provided	1
**Abstract**	3a	The rationale/justification of what the investigation contributes to the literature and/or addresses a gap in knowledge must be provided	5
	3b	The aim/objectives of the investigation must be provided	2, 5, 6
	3c	The body of the Abstract must describe the materials and methods used in the investigation and include information on data management and statistical analysis	2
	3d	The body of the Abstract must describe the most significant scientific results for all experimental and control groups	2
	3e	The main conclusion(s) of the study must be provided	2
**Introduction**	4a	A background summary of the scientific investigation with relevant information must be provided	4-5
	4b	The aim(s), purpose(s) or hypothesis(es) of an investigation must be provided ensuring they align with the methods and results	5-6
**Materials and Methods**	5a	A clear ethics statement and the ethical approval granted by an ethics board, such as an Institutional Review Board or Institutional Animal Care and Use Committee, must be described	7
	5b	When harvesting cells and tissues for research, all the legal, ethical, and welfare rights of human subjects and animal donors must be respected and applicable procedures described	7
	5c	The use of reference samples must be included, as well as negative and positive control samples, and the adequacy of the sample size justified	9
	5d	Sufficient information about the methods/materials/supplies/samples/specimens/instruments used in the study must be provided to enable it to be replicated	7-8
	5e	The use of categories must be defined, reliable and be described in detail	7-9
	5f	The numbers of replicated identical samples must be described within each test group. The number of times each test was repeated must be described	9
	5g	The details of all the sterilization, disinfection, and handling conditions must be provided, if relevant	Not applicable
	5h	The process of randomization and allocation concealment, including who generated the random allocation sequence, who decided on which specimens to be included and who assigned specimens to the intervention must be provided(if applicable)	Not applicable
	5i	The process of blinding the operator who is conducting the experiment (if applicable) and the examiners when assessing the results must be provided	8

	5j	Information on data management and analysis including the statistical tests and software used must be provided	9
**Results**	6a	The estimated effect size and its precision for all the objective (primary and secondary) for each group including controls must be provided	10
	6b	Information on the loss of samples during experimentation and the reasons must be provided, if relevant	Not applicable
	6c	All the statistical results, including all comparisons between groups must be provided	10-11
**Discussion**	7a	The relevant literature and status of the hypothesis must be described	12, 13
	7b	The true significance of the investigation must be described	12-13
	7c	The strength(s) of the study must be described	12-13
	7d	The limitations of the study must be described	13
	7e	The implications for future research must be described	14
**Conclusion(s)**	8a	The rationale for the conclusion(s) must be provided	10-11
	8b	Explicit conclusion(s) must be provided, i.e. the main "take-away" lessons	14
**Funding and support**	9a	Sources of funding and other support (such as supply of drugs, equipment) as well as the role of funders must be acknowledged and described	2
**Conflicts of interest**	10a	An explicit statement on conflicts of interest must be provided	2-3
**Quality of images**	11a	Details of the relevant equipment, software and settings used to acquire the image(s) must be described in the text or legend	8
	11b	If an image(s) is included in the manuscript, the reason why the image(s) was acquired and why it is included must be provided in the text	Not applicable
	11c	The circumstances (conditions) under which the image(s) were viewed and evaluated must be provided in the text	Not applicable
	11d	The resolution and any magnification of the image(s) or any modifications/ enhancements (e.g. brightness, image smoothing, staining etc.) that were carried out must be described in the text or legend	Not applicable
	11e	An interpretation of the findings (meaning and implications) from the image (s) must be provided in the text	12-13
	11f	The legend associated with each image must describe clearly what the subject is and what specific feature(s) it illustrates	30, 32
	11g	Markers/labels must be used to identify the key information in the image(s) and defined in the legend	19-21, 30
	11h	If relevant, the legend of each image must include an explanation whether it is pre-experiment, intra-experiment or post-experiment and, if relevant, how images over time were standardised	Not applicable

From: Nagendrababu V, Murray PE, Ordinola-Zapata R, Peters OA, Rôças IN, Siqueira JF Jr, Priya E, Jayaraman J, Pulikkotil SJ, Camilleri J, Boutsioukis C, Rossi-Fedele G, Dummer PMH (2021) PRILE 2021 guidelines for reporting laboratory studies in Endodontology: a consensus-based development.** ***International Endodontic Journal **May 3. doi: 10.1111/iej.13542. https://onlinelibrary.wiley.com/doi/abs/10.1111/iej.13542**

For further details visit: http://pride-endodonticguidelines.org/prile

Table S2. Absorbance determined by the MTT assay after 7 days of incubation with bioceramic materials.

	**1**	**2**	**3**	**4**	**5**	**Mean (SD)**
HP	1.85	0.88	1.30	1.27	0.99	1.26 (0.39)
BG	1.11	1.16	1.03	1.27	0.61	1.04 (0.25)
F	0.51	0.72	0.81	0.33	0.16	0.50 (0.27)
NC	1.57	1.58	1.47	1.47	1.47	1.51 (0.06)

HP: MTA Repair HP; F: MTA Flow White; BG: Nishika Canal Sealer BG multi; NC: negative control.

Table S3. Absorbance determined by the MTT assay after 28 days of incubation with MTA Flow White in different powder-liquid ratios.

	**1**	**2**	**Mean (SD)**
F0	0.01	0.01	0.01 (0.00)
F1	0.56	1.22	0.89 (0.46)
F2	0.02	3.16	1.59 (2.22)
NC	1.33	1.19	1.26 (0.10)

F0, F1 and F2: MTA Flow White mixed in three different consistencies: F0 (0.19 g powder per 0.12 g liquid), F1 (0.26 g powder per 0.12 g liquid), and F2 (0.19 g powder per 0.04 g liquid); NC: negative control.

Table S4. Absorbance determined by the MTT assay after 28 days of incubation with Nishika Canal Sealer BG multi in different powder-paste ratios.

	**1**	**2**	**Mean (SD)**
BG0	1.00	0.39	0.69 (0.43)
BG1	1.23	1.66	1.44 (0.30)
BG2	1.08	2.03	1.55 (0.67)
NC	1.56	1.73	1.64 (0.12)

BG0, BG1 and BG2: Nishika Canal Sealer BG multi mixed in three different consistencies: BG0 (no powder added), BG1 (0.03 g powder per 0.09 g paste), and BG2 (0.06 g powder per 0.09 g paste); NC: negative control.

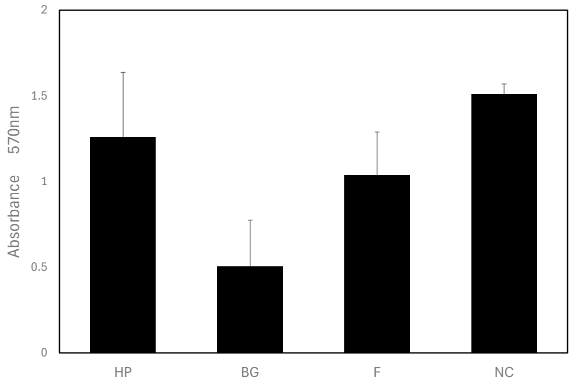

Figure S1. Cytocompatibility of endodontic bioceramics in hPDLCs. After 7 days of incubation with bioceramic materials. HP: MTA Repair HP; BG: Nishika Canal Sealer BG multi; F: MTA Flow White; NC: negative control.

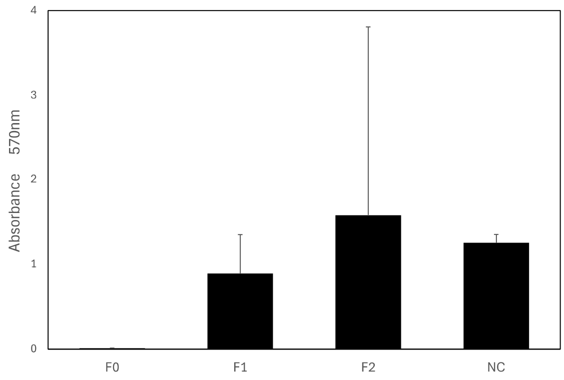

Figure S2. Cytocompatibility of MTA Flow White in different powder-liquid (paste) ratio with hPDLCs. After 28 days of incubation with MTA Flow White mixed in three different consistencies: F0 (0.19 g powder per 0.12 g liquid), F1 (0.26 g powder per 0.12 g liquid), and F2 (0.19 g powder per 0.04 g liquid). F: MTA Flow White; NC: negative control.

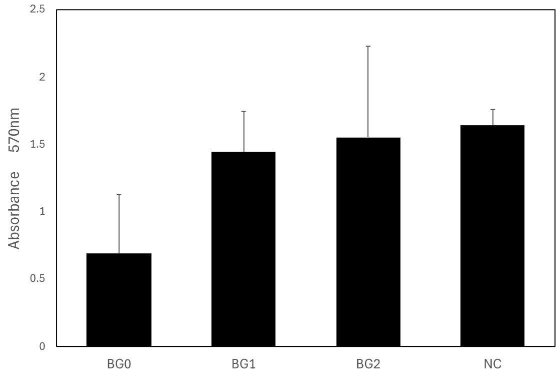

Figure S3. Cytocompatibility of Nishika Canal Sealer BG multi in different powder-liquid (paste) ratio with hPDLCs. After 28 days of incubation with Nishika Canal Sealer BG multi mixed in three different

consistencies: BG0 (no powder added), BG1 (0.03 g powder per 0.09 g paste), and BG2 (0.06 g powder per 0.09 g paste). F: MTA Flow White; NC: negative control.
